# Tumor Microenvironment-Associated Extracellular Matrix Components Regulate NK Cell Function

**DOI:** 10.3389/fimmu.2020.00073

**Published:** 2020-01-29

**Authors:** Gustavo Rodrigues Rossi, Edvaldo S. Trindade, Fernando Souza-Fonseca-Guimaraes

**Affiliations:** ^1^Cellular Biology Department, Federal University of Paraná, Curitiba, Brazil; ^2^Diamantina Institute, Translational Research Institute, University of Queensland, Brisbane, QLD, Australia

**Keywords:** NK cells, tumor microenvironment, galectin, MMP, ADAM

## Abstract

The tumor microenvironment (TME) is composed of multiple infiltrating host cells (e.g., endothelial cells, fibroblasts, lymphocytes, and myeloid cells), extracellular matrix, and various secreted or cell membrane-presented molecules. Group 1 innate lymphoid cells (ILCs), which includes natural killer (NK) cells and ILC1, contribute to protecting the host against cancer and infection. Both subsets are able to quickly produce cytokines such as interferon gamma (IFN-γ), chemokines, and other growth factors in response to activating signals. However, the TME provides many molecules that can prevent the potential effector function of these cells, thereby protecting the tumor. For example, TME-derived tumor growth factor (TGF)-β and associated members of the superfamily downregulate NK cell cytotoxicity, cytokine secretion, metabolism, proliferation, and induce effector NK cells to upregulate ILC1-like characteristics. In concert, a family of carbohydrate-binding proteins called galectins, which can be produced by different cells composing the TME, can downregulate NK cell function. Matrix metalloproteinase (MMP) and a disintegrin and metalloproteinase (ADAM) are also enzymes that can remodel the extracellular matrix and shred receptors from the tumor cell surface, impairing the activation of NK cells and leading to less effective effector functions. Gaining a better understanding of the characteristics of the TME and its associated factors, such as infiltrating cells and extracellular matrix, could lead to tailoring of new personalized immunotherapy approaches. This review provides an overview of our current knowledge on the impact of the TME and extracellular matrix-associated components on differentiation, impairment, and function of NK cells.

## Introduction

Innate lymphoid cells (ILCs) are lymphocytes derived from a common lymphoid precursor. Unlike T and B lymphocytes, ILCs do not express adaptive antigen receptors, instead being activated through cytokine receptors (1). Natural killer (NK) cells are classified as group 1 ILCs together with ILC1 (2), previously known as tissue resident NK cells (3), due to their shared dependence on the transcription factor T-bet, production of specific cytokines (e.g., interferon-gamma, IFN-γ), and surface receptor expression (e.g., NK1.1, NKp46 in mice, and NKp30 in humans) (1, 4). During development in the bone marrow, both mouse and human NK cells appear dependent on the transcription factor Eomesdermin (Eomes) (5), then during maturation they repress Eomes and increase T-bet production (4). However, in humans, mature liver ILC1s can express Eomes (6). Although ILC1 are tissue resident and unlikely to migrate to other tissues (7–9), their function in cancer is poorly understood. Recent studies have revealed that transforming growth factor-beta (TGF-β) signaling (either by TGF-β itself or indirectly by Activin-A) can suppress cellular metabolism and effector functions (10–12). This suppressive signaling drives the upregulation of ILC1-related markers in circulating mouse or human NK cells, suggesting the possibility of intercellular plasticity which could be important within the tumor microenvironment (TME) (11, 13, 14). NK cell cytotoxicity can be controlled by many stimulatory (NKp30, NKp44, NKp46, CD16) and inhibitory (PD1, TIM3, TIGIT, KLRG1) surface receptors (15). Even with their ability to kill transformed cells, NK cell immunosurveillance can be evaded by tumor cells due to their ability to manipulate the TME in favor of immune equilibrium and escape, allowing tumor survival and the possibility of further metastatic spread (16–18). Once established, the TME is composed of different immune and non-immune cell subsets recruited by the tumor (e.g., fibroblasts, pericytes, endothelial cells, macrophages, lymphocytes, etc.) (19), bioactive products, such as extracellular matrix (ECM) proteins, cytokines, and growth factors (20), and specific glycosylation pattern (21, 22). In this review we will discuss some of the molecules present in the TME (summarized on Table 1), with a focus on their potential impact on NK cell functions.

**Table 1:** TME molecules and the effect on NK cells.

**TME molecule**	**Effect on NK cells**	**Cancer type**	**References**
Hyaluronan	Impair access to tumor and ADCC	Breast and ovarian caner	(23)
Heparanase	Decrease recognition of target cells	Breast cancer	(24)
Galectin-1	Impair cytotoxicity	Glioma	(25)
Galectin-3	Galectin-3^−/−^ mice have more effective cytotoxic CD27^high^CD11b^high^ NK cells	Melanoma	(26)
	Increase of galectin-3 impair NK cells cytotoxicity	Adenocarcinoma, cervix cancer	(27)
Galectin-9	Increase of NK cells infiltration	Melanoma	(28)
	Downregulation of stimulatory genes (LTB, KLRF1, FCGR3A) and impair cytotoxicity against K562 cells	Leukemia	(29)
	Galectin-9 binds to TIM-3 leading to NK cells exhaustion	Gastrointestinal tumors	(30)
Sialic acid	Low sialylation of tumor cells increases NK cell cytotoxicity	Melanoma	(31)
Siglec-7/9	Membrane inhibitory receptor on NK cells that recognize sialic acid	Melanoma, basal cell carcinoma, squamous cell carcinoma, and cutaneous T cell lymphoma	(32)
MMP-9	Cleaves MIC-A, MIC-B and ULBP-2 from tumor cells membrane avoiding killing by NK cells	Human gastric cancer, lung adenocarcinoma and osteosarcoma	(33–35)
ADAM-10/17	Cleave MIC-B, ULBP-2 and B7-H6 from tumor cells membrane avoiding killing by NK cells	Human pancreatic adenocarcinoma, melanoma, cervical, breast, hepatocellular carcinomas and glioblastoma	(36–38)

## Glycosaminoglycans and Proteoglycans

Glycosaminoglycans (GAGs) are a family of linear polysaccharides composed of repeating disaccharide units. Depending on the disaccharide composition, GAGs can be classified as: keratan, chondroitin, dermatan or heparan sulfate (HS), heparin, or hyaluronan (39). Except for hyaluronan, all GAGs can be linked to proteins, forming proteoglycans (PGs) (40).

Hyaluronan is the only non-sulfated GAG, first isolated and characterized from bovine vitreous humor in 1934 (41). It is produced and secreted to the ECM by the transmembrane hyaluronan synthase (42), which is encoded by three conserved genes in both mice (*Has1, Has2*, and *Has3*) and humans (*HAS1, HAS2*, and *HAS3*) (43, 44). In cancer, hyaluronan is associated with tumor cell proliferation, angiogenesis, and evasion of immune responses and apoptosis (45–50). The presence of hyaluronan in the TME appears to be detrimental to NK cell function against cancer cells; hyaluronan rich tumors can inhibit both NK cell access to tumor cells and antibody-dependent cell-mediated cytotoxicity (ADCC) (23). Although hyaluronan does not form PGs, it can bind to PGs by linking proteins (51). Our group recently identified a poor prognostic association between the *HAPLN3* gene (Hyaluronan and Proteoglycan Link Protein 3) and a low NK cell infiltration in malignant melanoma patients, suggesting a potential inhibition of anti-tumor immune functions by *HAPLN3* and identifying this gene as a potential target for immunotherapy (52).

Heparan sulfate proteoglycans (HSPGs) can be found on the cell surface (glypicans and syndecans families) or in the ECM (perlecan, agrin, collagen XVIII) (53). Many types of tumors overexpress HSPGs, which is associated with increased angiogenesis in hepatocellular and colon carcinomas, breast and pancreatic cancers, and melanoma (54–58). HSPGs are also associated with invasion and metastasis in melanoma and breast cancer (59–61). Some reports have suggested that HS chains can be ligands for NKp30 (62, 63), NKp44 (63, 64), NKp46 (62, 63, 65), and for the NKG2D and CD94 complex (66). This tumor production of HSPG is not sufficient to stimulate NK cell cytotoxicity, and there are two potential hypotheses for this observation:

- Tumor cells present altered expression of many enzymes related to the HSPG modifications, such as sulfatase 2 and heparan sulfate 6-O- sulfotransferase 2 (67–69), leading to production of PGs containing distinctly sulfated HS chains (70, 71). Differences in sulfation pattern could impair the recognition of HS chains by NKp30, NKp44, and NKp46 (62, 63, 65).
- Melanomas, multiple myeloma, bladder, prostate, breast, colon and liver cancers overexpress heparanase (72–76), which is an endo β-D-glucuronidase that cleaves specific regions of HS into small fragments (77, 78), decreasing NK cells ability to recognize target cells (24). However, a previous study showed that heparanase produced by NK cells is also unexpectedly important for the host tumor surveillance by allowing NK cell navigation through the ECM (79).

## Galectins

Galectins are a group of proteins with two main features: β-galactoside binding sites and conserved carbohydrate recognition domains (CRDs) (80). The first galectin was isolated in 1975 from an electric fish (*Electrophorus electricus*) and named electrolectin (81). Just in 1994, the name galectin was given to this family of lectins and all members were numbered in order of discovery (80). Galectins are divided into three groups: prototype have one CRD domain (galectins 1, 2, 7, 10, 11, 13, 14, and 15); tandem-repeat type have two CDR domains (galectins 4, 8, 9, and 12); chimera-type have a single CRD domain and an amino-terminal polypeptide rich in proline, glycine, and tyrosine residues (galectin-3) (82).

Galectins are expressed in many different mammalian tissues (83, 84) and are involved in early development, tissue regeneration, immune homeostasis, and some pathologies (e.g., cancer, obesity, type II diabetes) (85). In some types of cancer, galectins may be associated with angiogenesis, cancer cell survival, invasion, metastasis, and avoiding immunosurveillance (86). Here we will discuss and revisit the potential contribution of different galectins for the TME, NK cell function, and anti-cancer responses.

Galectin-1 is important for maturation of B cells in the bone marrow (87, 88) and T cells homeostasis (89–91). It is overexpressed in some types of cancer such as ovarian, breast, myeloma, and melanoma (92–95), and can contribute to tumor survival by inhibition of NK cells (25). Glioma cells deficient for galectin-1 showed reduced tumor growth, increased intra-tumor NK cell infiltration, and elevated expression of granzyme B when implanted into the striatum of *Rag1*^−/−^ mice (which develop NK, but not T or B cells) when compared to *Rag1*^−/−^ mice injected with wild-type cells (25). In the same study, galectin-1 deficient glioma cells were injected into NGS (T, B, and NK cells deficient) or C57BL/6 immunocompetent mice treated with anti-asialo GM1, which depletes NK cells. Enhanced tumor growth was observed in both models, proving the inhibitory effect galectin-1 has on NK cell anti-tumor function (25).

Galectin-3 was initially discovered in macrophages and named Mac-2 (96). It starts to be expressed in many normal tissues during embryogenesis (in both mice and humans) (97) and is involved in angiogenesis (98) and migration of monocytes and macrophages (99). In cancer, galectin-3 overexpression in the TME is associated with angiogenesis (98), tumor progression (97), and immune escape by inducing T cell apoptosis (100, 101). Some reports have also shown the impact of galectin-3 on NK cells. For example, galetin-3-deficient mice are resistant to lung metastasis development by B16-F1 melanoma cells, potentially due to an increase of CD27^high^ CD11b^high^ NK cells in their spleen compared with the wild type (26), suggesting an inhibitory effect of galectin-3 on NK cell immunosurveillance. Additionally, HeLa cells overexpressing galectin-3 are more resistant to human NK cell-mediated death; yet when galectin-3 is knocked out the killing capacity of NK cells is restored in a mechanism mainly mediated by NKp30 (27). Considering the potential for galectins as cancer treatment targets, clinical trials using galectin inhibitors have already started for both galectins 1 (ClinicalTrials.gov identifier: NCT01724320—for advanced solid tumors; NCT00054977—for advanced solid tumors in combination or not with 5-Fluorouracil) and 3 (NCT02575404—for advanced melanoma, non-small cell lung cancer, and head and neck squamous cell cancer in combination with Pembrolizumab; NCT02117362—for advanced melanoma in combination with Ipilimumab).

Galectin-9 was first described in mouse embryos and later discovered during homeostasis in many adult organs such as liver, kidney, spleen, and lungs (102). In some cancers, galectin-9 is related with a good prognosis (103). In breast, pancreatic cancer and melanoma, expression of galectin-9 correlates with good prognosis for those patients (28, 104, 105). Galectin-9 appears to promote patient survival in part through NK cell modulation (106). C57BL/6 mice that had B16-F10 melanoma injected into their peritoneal cavity followed by galectin-9 treatment showed prolonged survival compared with untreated controls, which also correlated with increased NK cell infiltration into the peritoneal cavity; however, when NK cells were depleted by anti-asialo GM1, those positive effects were lost, suggesting a stimulatory effect of galectin-9 on NK cells (106). Despite these findings, the role of galectin-9 may be ambiguous, as inhibitory effects over NK cells have also been demonstrated (29). Human NK cells exposed to galectin-9 downregulate many NK cell stimulatory genes (e.g., LTB, KLRF1, FCGR3A), resulting in less efficient killing of target leukemia K562 cells (29). A possible explanation for galectin-9 mediated inhibition of NK cells could be its interaction with and activation of TIM-3 (T cell immunoglobulin and mucin domain 3) (107), which is a transmembrane receptor associated with NK cell exhaustion (108, 109). Additionally, a positive correlation was found between galectin-9 expression on human gastrointestinal stromal tumor and TIM-3^+^ expression on infiltrating NK cells (30). This study suggests that targeting galectin-9 or preventing its interaction with TIM-3 could potentially act as a novel immunotherapy approach to enhance NK cell functions against cancer (30).

## Sialic Acid and Mucins

Sialic acids (Sia) are a family of carbohydrates composed of *N*-acetylneuraminic acids (110) linked to many proteins, lipids, and other polysaccharides on the cell surface. The most common Sia are *N*-acetylneuraminic (Neu5Ac) and *N*-glycolylneuraminic (Neu5Gc) acids (111). Humans only express Neu5Ac, due to the lack of an enzyme called cytidine monophospho-*N*-acetylneuraminic acid hydroxylase, which converts Neu5Ac to Neu5Gc (112). Sia are associated with many biological processes, but an important function is recognizing self and non-self (113). Many types of cancer, including breast cancer and cervix squamous cell carcinoma, are hypersialyated (114, 115) due to the overexpression of Sia synthesis enzymes (116, 117). This hypersialytion is associated with increased metastasis (117) and immune system evasion (118). A study using “Sia low” B16-F10 cells demonstrated that after their subcutaneous injection into C57BL/6 mice, tumors grew more slowly and exhibited increased NK cell infiltration when compared with standard B16-F10 cells (31). Additionally, after NK cell depletion (using anti-NK1.1) “low Sia” tumors grew at a similar rate to the control group, highlighting the importance of NK cells during the defense against sialyated tumors (31).

**Figure 1:**
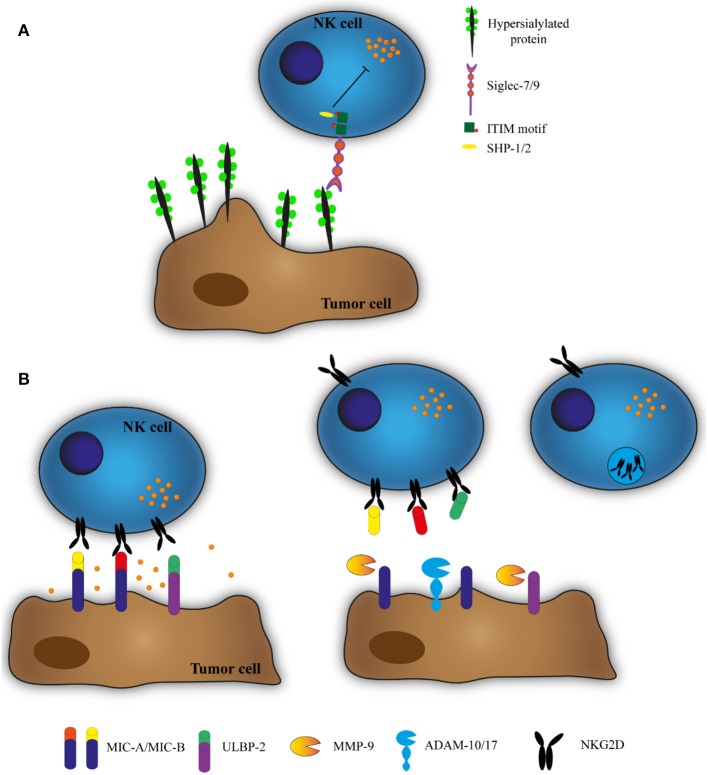
**(A)** Hypersialysation of tumor cells inhibits NK cell cytotoxicity. To impair recognition by NK cells, tumor cells change their glycosylation pattern, expressing more sialic acid on the cell membrane. NK cells express membrane receptors that recognize this sialic acid (Siglecs). Siglecs have an intracellular immune receptor tyrosine-based inhibition motif (ITIM) that recruits tyrosine phosphatases SHP-1 and SHP-2 and inhibits NK cell cytotoxicity. **(B)** ADAMs and MMPs cleave MIC-A, MIC-B, and ULBP-2 and downregulate NKG2D expression. NK cells can recognize and kill target cells by the interaction between the stimulatory receptor NKG2D and the ligands MIC-A, MIC-B, and ULBP-2. However, the TME contains ADAMs and MMPs that cleave these ligands, allowing the soluble proteins to bind to NKG2D and stimulate its degradation.

The interactions between cells and Sia are mediated by transmembrane proteins called Siglecs (sialic acid-binding immunoglobulin-type lectins) (119). Siglecs are expressed in all immune cells and are divided into two broad groups: CD33 and CD33-related Siglecs, which have high homology with CD33 in their extracellular domains, and CD33-unrelated Siglecs which have high homology between human, rodents and other vertebrates (120). Both groups consist of both activating and inhibitory receptors, where the inhibitory Siglecs contain the intracellular immune receptor tyrosine-based inhibition motifs (ITIM), leading to tyrosine phosphorylation and tyrosine phosphatases SHP-1 and SHP-2 (121) (and as exemplified in Figure 1A). ITIMs are associated with NK cell inhibition and are related to other inhibitory receptors (e.g., Ly-49 and NKG2-A) (122, 123). Human NK cells express Siglec-7 (also named as p75/AIRM1) (124, 125) and Siglec-9 (126) on the cell surface. Siglec-7 is expressed in all human NK cells (124, 125) whereas Siglec-9 is expressed selectively in a subset of CD56^dim^ NK cells (32, 127). Jandus and colleagues demonstrated that various human tumor samples (melanoma, basal cell carcinoma, squamous cell carcinoma, and cutaneous T cell lymphoma) and tumor cell lines (e.g., A375, HeLa, SW1116, and K562) have ligands for both Siglec-7 and 9. They also found that NK cells displayed increased cytotoxicity against HeLa and K562 after enzymatic treatment to remove the Sia from the target cell surface (32). Cell lines of multiple myeloma (e.g., RPMI 8226 and H929) pre-treated with a sialyltransferase inhibitor were also more susceptible to NK cell-mediated killing (128). In a separate study, Balb/c mice injected with desialylated MCA-induced fibrosarcoma cells developed less lung metastasis, an effect which could be abolished when NK cells were depleted by antibodies (129). Besides inhibitors of sialytranferase and enzymes that cleave Sia, other strategies can be applied to avoid Sia-mediated inhibition of NK cells, and antagonists for Siglecs-7 and 9 could be an option (130). This has been demonstrated by Prescher and collaborators, who described a small molecule inhibitor of Siglec-7 which increased cytotoxicity of human NK cells toward Mel1106 melanoma target cells (130).

Siglec-9 can also interact with mucin-1 and 16 (127, 131), which are rich in Sia (132, 133). Mucins are proteins that have tandem repeat structures which are highly glycosylated and rich in proline, threonine, and serine (PTS domains) (134). They are normally expressed by epithelial cells, but are overexpressed in some types of cancer, particularly ovarian (135). Some reports have shown that murine ovarian cancer cells knocked down for mucin-16 are more susceptible for NK cell killing, showing that mucin-16 has an impact on NK cells (136, 137). While mucin-1 is also a ligand for Siglec-9, it has only been demonstrated to have a direct inhibition on macrophages (138). However, mucin-1 may have other effects on NK cells (139). In human metastatic bladder cancer, tumor cells overexpress the enzyme 2β-1,6-N-acetylglucosaminyltransferase (C2GnT) that adds a poly-N-acetyllactosamine on Mucin-1. The increased glycosylation of Mucin-1 raises its affinity for galectin-3 binding. Consequently, this Mucin-1/galectin-3 complex is suggested to generate a shield around tumor cells, which impairs recognition by NK cells (139).

## Matrix Metalloproteinases (MMPs) and A Disintegrin and Metalloproteinases (ADAMs)

Matrix metalloproteinases (MMPs) and a disintegrin and metalloproteinases (ADAMs) belong to a superfamily of zinc-dependent metalloproteinases known as metzincins, which process or degrade virtually all structural ECM proteins, growth factor–binding proteins, cell-cell adhesion molecules, and cell surface receptors (138, 139). MMPs are found either on the cell surface or soluble, and are involved in tissue remodeling and wound healing (140). ADAMs are single-pass membrane proteins that are important in shedding proteins and embryogenesis (141). In many types of cancer, MMPs and ADAMs are associated with tumor progression through angiogenesis, invasion, metastasis, and regulation of the immune response (142, 143).

MMPs and ADAMs can cleave NKG2D ligands from the tumor cell surface, including MHC class I chain-related A (MIC-A), MHC class I chain-related B (MIC-B), and UL16-binding protein (ULBP) (144, 145). The soluble forms of cleaved proteins from tumor cell membrane bind to NKG2D, inducing endocytosis and degradation of this receptor, resulting in the tumor evasion from the surveillance of this receptor (144, 146) (Figure 1B). This effect has been observed in multiple studies using different tumor cell lines, and in all of them the NK cell function returns to normal after using inhibitors for MMPs or ADAMs (33–37). *Ferrari de Andrade* and collaborators developed an antibody that binds to the MIC-A α3 domain, the site of proteolytic shedding, to avoid MIC-A cleavage, and demonstrated this could increase NK cell cytotoxicity toward human melanoma cells (147).

MMPs can also shed intercellular-adhesion molecule 1 (ICAM-1) from the tumor cell surface, a protein that is important for the adhesion of cytotoxic T lymphocytes and NK cells to target cells (148, 149). Interaction of NK cells with target cells expressing ICAM-1 leads to an expression of IFN-γ (150). Many types of cancers express ICAM-1 (151), however it is thought to be shed from the surface of tumor cells to avoid an immune response (152, 153). Indeed, when comparing the human breast cancer cell line MDA-MB435 (ICAM-1^+^ and MMP-9^−^) to transfected MDA-MB435 (ICAM-1^+^ and MMP-9^+^), the transfected cells had a higher concentration of soluble ICAM-1 in the supernatant and were more resistant to NK cells. This resistance was reversed when those cells were co-cultured in the presence of MMP-9 inhibitors (154).

ADAM-10 and 17 can also catalyze the cleavage of B7-H6, one of the ligands for NKp30 (both only expressed in human) (38). Using many different human tumor cell lines (pancreatic adenocarcinoma, melanoma, cervical, breast, and hepatocellular carcinomas), Schlecker and colleagues observed that these cells produced B7-H6 at the mRNA level; however they had a low abundance of this protein on the cell membrane compared to what was detectable in the culture supernatant, showing ADAM-10 and 17 cleaving activity (38). The high levels of soluble B7-H6 decreased the expression of NKp30 on the NK cell membrane, leading to a decrease of degranulation. However, in the presence of inhibitors or siRNA for ADAM-10 or 17, the levels of soluble B7-H6 decreased and the degranulation of NK cells was restored (38). Curiously, several reports have also described the effects of ADAM-17 in cleaving CD16 (FcgRIIIA), one of the most important activating receptors responsible for recognition of antibody-coated target cells and NK cell-mediated ADCC, suggesting the potential for inhibitors of ADAM17 as a novel therapeutic approach to increase NK cell anti-tumor potency during immunotherapy (155). As an alternative to prevent ADAM-17-mediated shedding of CD16, Jing and colleagues showed that replacing the serine at position 197 of the cleavage site of CD16 with proline completely prevented ADAM-17-mediated cleavage of both CD16a and b, enhancing NK cell function to antibody-opsonized tumor cells (156). More recently, the same group provided evidence that amino acid replacement to generate uncleavable CD16 can be feasibly employed in induced human pluripotent stem cells (hiPSC), as a renewable and gene-editable source of off-the-shelf NK cell products with enhanced functionality (157).

Peng and collaborators showed that MMPs can also have a direct effect on NK cells, leading to their dysfunction. NK cells were co-cultured with a pancreatic cancer cell line (SW1990), and an increase of MMP-9 production was observed compared with NK cells co-cultured with a normal pancreatic cell line (hTERT-HPNE) (158). It was also observed that NK cells after been co-cultured with SW1990 presented a reduction in the percentage of cells positive for NKG2D, NKp30, NKp44, NKp46, DNAM-1, perforin, and granzyme B, and those cells were less cytotoxic against K563 (158). However, after incubation with an inhibitor for MMP-9 (TIMP-1) the levels of NKG2D, NKp30 and perforin were partially restored and the killing capacity was recovered (158). Additionally, in concert with our previous observations in murine NK cells (13), Bruno and colleagues described that infiltrating NK cells in human colorectal tumors display a “decidual” behavior by expression of CD49a (among other tissue resident-related markers) and MMP-9 (159). The same study also revealed MMP-9-expressing NK cells as important contributors of tumor angiogenesis, and that inhibition of MMP-9 with immunotherapy could help repolarize NK from pro-angiogenesis to anti-tumor effector cells (159). These recent findings reveal that MMPs might not only play a role in NK cell migration and *in vivo* positioning as previously believed (160), but also directly impact their anti-tumorigenic function and potentially be considered as novel inhibitory checkpoints in NK cell biology.

## Conclusion

Many components of the TME can impair the cytotoxic activity of NK cells by changing or cleaving ligands that could lead the activation of NK cells, or by an increasing the availability of factors that can downregulate NK cells effector functions. There is an arising interest for identifying novel immune checkpoints for NK cells. Studies around the composition of the TME, such as ECM proteins, enzymes, and glycosylation patterns, are now a field of interest to understand how to overcome tumor inhibitory signals and discover new therapeutic targets.

## Author Contributions

GR and FS-F-G wrote the manuscript. ET and FS-F-G reviewed the manuscript and provided critical input.

### Conflict of Interest

The authors declare that the research was conducted in the absence of any commercial or financial relationships that could be construed as a potential conflict of interest.

## References

[1] Vivier E Artis D Colonna M Diefenbach A Di Santo JP Eberl G. Innate lymphoid cells: 10 years on. Cell. (2018) 174:1054–66. 10.1016/j.cell.2018.07.017 30142344

[2] Spits H Artis D Colonna M Diefenbach A Di Santo JP Eberl G. Innate lymphoid cells-a proposal for uniform nomenclature. Nat Rev Immunol. (2013) 13:145–9. 10.1038/nri3365 23348417

[3] Sojka DK Plougastel-Douglas B Yang L Pak-Wittel MA Artyomov MN Ivanova Y. Tissue-resident natural killer (NK) cells are cell lineages distinct from thymic and conventional splenic NK cells. Elife. (2014) 3:1–21. 10.7554/eLife.01659 24714492 PMC3975579

[4] Zhang J Marotel M Fauteux-Daniel S Mathieu A-L Viel S Marçais A. T-bet and Eomes govern differentiation and function of mouse and human NK cells and ILC1. Eur J Immunol. (2018) 48:738–50. 10.1002/eji.201747299 29424438

[5] Daussy C Faure F Mayol K Viel S Gasteiger G Charrier E. T-bet and Eomes instruct the development of two distinct natural killer cell lineages in the liver and in the bone marrow. J Exp Med. (2014) 211:563–77. 10.1084/jem.20131560 24516120 PMC3949572

[6] Aw Yeang HX Piersma SJ Lin Y Yang L Malkova ON Miner C. Cutting edge: human CD49e—NK cells are tissue resident in the liver. J Immunol. (2017) 198:1417–22. 10.4049/jimmunol.1601818 28093522 PMC5296254

[7] Gasteiger G Fan X Dikiy S Lee SY Rudensky AY Tissue residency of innate lymphoid cells in lymphoid and non-lymphoid organs. Science. (2015) 350:981–5. 10.1126/science.aac9593 26472762 PMC4720139

[8] Tang L Peng H Zhou J Chen Y Wei H Sun R. Differential phenotypic and functional properties of liver-resident NK cells and mucosal ILC1s. J Autoimmun. (2016) 67:29–35. 10.1016/j.jaut.2015.09.004 26422992

[9] Yokoyama WM Sojka DK Peng H Tian Z. Tissue-resident natural killer cells. Cold Spring Harb Symp Quant Biol. (2013) 78:149–56. 10.1101/sqb.2013.78.020354 24584057

[10] Viel S Marçais A Guimaraes FS-F Loftus R Rabilloud J Grau M. TGF-β inhibits the activation and functions of NK cells by repressing the mTOR pathway. Sci Signal. (2016) 9:ra19. 10.1126/scisignal.aad1884 26884601

[11] Rautela J Dagley LF de Oliveira CC Schuster IS Hediyeh-Zadeh S Delconte RB. Therapeutic blockade of activin-A improves NK cell function and antitumor immunity. Sci Signal. (2019) 12:eaat7527. 10.1126/scisignal.aat7527 31455725

[12] Zaiatz-Bittencourt V Finlay DK Gardiner CM. Canonical TGF-β signaling pathway represses human NK cell metabolism. J Immunol. (2018) 200:3934–41. 10.4049/jimmunol.1701461 29720425

[13] Gao Y Souza-Fonseca-Guimaraes F Bald T Ng SS Young A Ngiow SF. Tumor immunoevasion by the conversion of effector NK cells into type 1 innate lymphoid cells. Nat Immunol. (2017) 18:1004–15. 10.1038/ni.3800 28759001

[14] Cortez VS Ulland TK Cervantes-Barragan L Bando JK Robinette ML Wang Q. SMAD4 impedes the conversion of NK cells into ILC1-like cells by curtailing non-canonical TGF-β signaling. Nat Immunol. (2017) 18:995–1003. 10.1038/ni.3809 28759002 PMC5712491

[15] Chiossone L Dumas P-Y Vienne M Vivier E Natural killer cells and other innate lymphoid cells in cancer. Nat Rev Immunol. (2018) 18:671–88. 10.1038/s41577-018-0061-z 30209347

[16] Krasnova Y Putz EM Smyth MJ Souza-Fonseca-Guimaraes F. Bench to bedside: NK cells and control of metastasis. Clin Immunol. (2017) 177:50–9. 10.1016/j.clim.2015.10.001 26476139

[17] Souza-Fonseca-Guimaraes F Cursons J Huntington ND. The emergence of natural killer cells as a major target in cancer immunotherapy. Trends Immunol. (2019) 40:142–58. 10.1016/j.it.2018.12.003 30639050

[18] Dunn GP Old LJ Schreiber RD. The three Es of cancer immunoediting. Annu Rev Immunol. (2004) 22:329–60. 10.1146/annurev.immunol.22.012703.104803 15032581

[19] Hanahan D Coussens LM. Accessories to the crime: functions of cells recruited to the tumor microenvironment. Cancer Cell. (2012) 21:309–22. 10.1016/j.ccr.2012.02.022 22439926

[20] De Palma M Biziato D Petrova T V. Microenvironmental regulation of tumour angiogenesis. Nat Rev Cancer. (2017) 17:457–74. 10.1038/nrc.2017.51 28706266

[21] Espinoza-Sánchez NA Götte M. Role of cell surface proteoglycans in cancer immunotherapy. Semin Cancer Biol. (2019). [Epub ahead of print]. 10.1016/j.semcancer.2019.07.012 31336150

[22] Rabinovich GA van Kooyk Y Cobb BA. Glycobiology of immune responses. Ann N Y Acad Sci. (2012) 1253:1–15. 10.1111/j.1749-6632.2012.06492.x 22524422 PMC3884643

[23] Singha NC Nekoroski T Zhao C Symons R Jiang P Frost GI. Tumor-associated hyaluronan limits efficacy of monoclonal antibody therapy. Mol Cancer Ther. (2015) 14:523–32. 10.1158/1535-7163.MCT-14-0580 25512619

[24] Mayes K Elsayed Z Alhazmi A Waters M Alkhatib SG Roberts M. BPTF inhibits NK cell activity and the abundance of natural cytotoxicity receptor co-ligands. Oncotarget. (2017) 8:64344–57. 10.18632/oncotarget.17834 28969075 PMC5610007

[25] Baker GJ Chockley P Yadav VN Doherty R Ritt M Sivaramakrishnan S. Natural killer cells eradicate galectin-1-deficient glioma in the absence of adaptive immunity. Cancer Res. (2014) 74:5079–90. 10.1158/0008-5472.CAN-14-1203 25038230 PMC4184887

[26] Radosavljevic G Jovanovic I Majstorovic I Mitrovic M Lisnic VJ Arsenijevic N. Deletion of galectin-3 in the host attenuates metastasis of murine melanoma by modulating tumor adhesion and NK cell activity. Clin Exp Metast. (2011) 28:451–62. 10.1007/s10585-011-9383-y 21442355

[27] Wang W Guo H Geng J Zheng X Wei H Sun R. Tumor-released galectin-3, a soluble inhibitory ligand of human NKp30, plays an important role in tumor escape from NK cell attack. J Biol Chem. (2014) 289:33311–9. 10.1074/jbc.M114.603464 25315772 PMC4246088

[28] Kageshita T Kashio Y Yamauchi A Seki M Abedin MJ Nishi N. Possible role of galectin-9 in cell aggregation and apoptosis of human melanoma cell lines and its clinical significance. Int J Cancer. (2002) 99:809–16. 10.1002/ijc.10436 12115481

[29] Golden-Mason L McMahan RH Strong M Reisdorph R Mahaffey S Palmer BE. Galectin-9 functionally impairs natural killer cells in humans and mice. J Virol. (2013) 87:4835–45. 10.1128/JVI.01085-12 23408620 PMC3624298

[30] Komita H Koido S Hayashi K Kan S Ito M Kamata Y. Expression of immune checkpoint molecules of T cell immunoglobulin and mucin protein 3/galectin-9 for NK cell suppression in human gastrointestinal stromal tumors. Oncol Rep. (2015) 34:2099–105. 10.3892/or.2015.4149 26239720

[31] Perdicchio M Cornelissen LAM Streng-Ouwehand I Engels S Verstege MI Boon L. Tumor sialylation impedes T cell mediated anti-tumor responses while promoting tumor associated-regulatory T cells. Oncotarget. (2016) 7:8771–82. 10.18632/oncotarget.6822 26741508 PMC4891003

[32] Jandus C Boligan KF Chijioke O Liu H Dahlhaus M Démoulins T. Interactions between Siglec-7/9 receptors and ligands influence NK cell–dependent tumor immunosurveillance. J Clin Invest. (2014) 124:1810–20. 10.1172/JCI65899 24569453 PMC3973073

[33] Shiraishi K Mimura K Kua L-F Koh V Siang LK Nakajima S. Inhibition of MMP activity can restore NKG2D ligand expression in gastric cancer, leading to improved NK cell susceptibility. J Gastroenterol. (2016) 51:1101–11. 10.1007/s00535-016-1197-x 27002316

[34] Le Maux Chansac B Misse D Richon C Vergnon I Kubin M Soria J-C. Potentiation of NK cell-mediated cytotoxicity in human lung adenocarcinoma: role of NKG2D-dependent pathway. Int Immunol. (2008) 20:801–10. 10.1093/intimm/dxn038 18441340

[35] Sun D Wang X Zhang H Deng L Zhang Y. MMP9 mediates MICA shedding in human osteosarcomas. Cell Biol Int. (2011) 35:569–74. 10.1042/CBI20100431 21143201

[36] Zingoni A Cecere F Vulpis E Fionda C Molfetta R Soriani A. Genotoxic stress induces senescence-associated ADAM10-dependent release of NKG2D MIC ligands in multiple myeloma cells. J Immunol. (2015) 195:736–48. 10.4049/jimmunol.1402643 26071561

[37] Wolpert F Tritschler I Steinle A Weller M Eisele G. A disintegrin and metalloproteinases 10 and 17 modulate the immunogenicity of glioblastoma-initiating cells. Neuro Oncol. (2014) 16:382–91. 10.1093/neuonc/not232 24327582 PMC3922520

[38] Schlecker E Fiegler N Arnold A Altevogt P Rose-John S Moldenhauer G. Metalloprotease-mediated tumor cell shedding of B7-H6, the ligand of the natural killer cell-activating receptor NKp30. Cancer Res. (2014) 74:3429–40. 10.1158/0008-5472.CAN-13-3017 24780758

[39] Esko JD Kimata K Lindahl U Proteoglycans and sulfated glycosaminoglycans. In: Varki A Cummings R Esko JD Freeze H Stanley P Bertozzi CR Hart G Marilynn E editors. Essentials of Glycobiology. New York, NY: Cold Spring Harbor (2017). 20301236

[40] Couchman JR Pataki CA. An introduction to proteoglycans and their localization. J Histochem Cytochem. (2012) 60:885–97. 10.1369/0022155412464638 23019015 PMC3527888

[41] Meyer K Palmer JW Polysaccharide of vitreous humor. J Biol Chem. (1934) 107:629–34.

[42] Fraser JRE Laurent TC Laurent UBG. Hyaluronan: its nature, distribution, functions and turnover. J Intern Med. (1997) 242:27–33. 10.1046/j.1365-2796.1997.00170.x 9260563

[43] DeAngelis PL Papaconstantiou J Wigel PH. Molecular cloning, identification, and sequence of the hyaluronan synthase gene from group a streptococcus pyogenes. J Biol Chem. (1993) 268:19181–4. 8366070

[44] Spicer AP Seldin MF Olsen AS Brown N Wells DE Doggett NA. Chromosomal localization of the human and mouse hyaluronan synthase genes. Genomics. (1997) 41:493–7. 10.1006/geno.1997.4696 9169154

[45] Caon I Bartolini B Parnigoni A Caravà E Moretto P Viola M. Revisiting the hallmarks of cancer: the role of hyaluronan. Semin Cancer Biol. (2019). [Epub ahead of print]. 10.1016/j.semcancer.2019.07.007 31319162

[46] Itano N Sawai T Miyaishi O Kimata K. Relationship between hyaluronan production and metastatic potential of mouse mammary carcinoma cells. Cancer Res. (1999) 59:2499–504. 10344764

[47] Morera DS Hennig MS Talukder A Lokeshwar SD Wang J Garcia-Roig M. Hyaluronic acid family in bladder cancer: potential prognostic biomarkers and therapeutic targets. Br J Cancer. (2017) 117:1507–17. 10.1038/bjc.2017.318 28972965 PMC5680466

[48] Kosaki R Watanabe K Yamaguchi Y. Overproduction of hyaluronan by expression of the hyaluronan synthase Has2 enhances anchorage-independent growth and tumorigenicity. Cancer Res. (1999) 59:1141–5. 10070975

[49] Liu N Gao F Han Z Xu X Underhill CB Zhang L. Hyaluronan synthase 3 overexpression promotes the growth of TSU prostate cancer cells. Cancer Res. (2001) 61:5207–14. 11431361

[50] Koyama H Hibi T Isogai Z Yoneda M Fujimori M Amano J. Hyperproduction of hyaluronan in Neu-induced mammary tumor accelerates angiogenesis through stromal cell recruitment: possible involvement of versican/PG-M. Am J Pathol. (2007) 170:1086–99. 10.2353/ajpath.2007.060793 17322391 PMC1864876

[51] Binette F Cravens J Kahoussi B Haudenschild DR Goetinck PF. Link protein is ubiquitously expressed in non-cartilaginous tissues where it enhances and stabilizes the interaction of proteoglycans with hyaluronic acid. J Biol Chem. (1994) 269:19116–22. 8034670

[52] Cursons J Souza-Fonseca-Guimaraes F Foroutan M Anderson A Hollande F Hediyeh-Zadeh S. A gene signature predicting natural killer cell infiltration and improved survival in melanoma patients. Cancer Immunol Res. (2019) 7:1162–74. 10.1158/2326-6066.CIR-18-0500 31088844

[53] Iozzo RV Schaefer L. Proteoglycan form and function: a comprehensive nomenclature of proteoglycans. Matrix Biol. (2015) 42:11–55. 10.1016/j.matbio.2015.02.003 25701227 PMC4859157

[54] Batmunkh E Schaff Z Kovalszky I Comparison of the expression of agrin, a basement membrane heparan sulfate proteoglycan, in cholangiocarcinoma and hepatocellular carcinoma B. Hum Pathol. (2007) 38:1508–15. 10.1016/j.humpath.2007.02.017 17640714

[55] Sharma B Handler M Eichstetter I Whitelock JM Nugent MA Iozzo R V. Antisense targeting of perlecan blocks tumor growth and angiogenesis *in vivo*. J Clin Invest. (1998) 102:1599–608. 10.1172/JCI3793 9788974 PMC509011

[56] Maeda T Desouky J Friedl A. Syndecan-1 expression by stromal fibroblasts promotes breast carcinoma growth *in vivo* and stimulates tumor angiogenesis. Oncogene. (2006) 25:1408–12. 10.1038/sj.onc.1209168 16247452

[57] Aikawa T Whipple CA Lopez ME Gunn J Young A Lander AD. Glypican-1 modulates the angiogenic and metastatic potential of human and mouse cancer cells. J Clin Invest. (2008) 118:89–99. 10.1172/JCI32412 18064304 PMC2117766

[58] Roy M Marchetti D. Cell surface heparan sulfate released by heparanase promotes melanoma cell migration and angiogenesis. J Cell Biochem. (2009) 106:200–9. 10.1002/jcb.22005 19115257 PMC2736788

[59] O'Connell MP Fiori JL Kershner EK Frank BP Indig FE Taub DD. Heparan sulfate proteoglycan modulation of Wnt5A signal transduction in metastatic melanoma cells. J Biol Chem. (2009) 284:28704–12. 10.1074/jbc.M109.028498 19696445 PMC2781415

[60] McFarlane S Coulter JA Tibbits P O'Grady A McFarlane C Montgomery N. CD44 increases the efficiency of distant metastasis of breast cancer. Oncotarget. (2015) 6:11465–76. 10.18632/oncotarget.3410 25888636 PMC4484469

[61] Adatia R Albini A Carlone S Giunciuglio D Benelli R Santi L. Suppression of invasive behavior of melanoma cells by stable expression of anti-sense perlecan cDNA. Ann Oncol. (1997) 8:1257–61. 10.1023/A:1008243115385 9496392

[62] Bloushtain N Qimron U Bar-Ilan A Hershkovitz O Gazit R Fima E. Membrane-associated heparan sulfate proteoglycans are involved in the recognition of cellular targets by NKp30 and NKp46. J Immunol. (2004) 173:2392–401. 10.4049/jimmunol.173.4.2392 15294952

[63] Hecht ML Rosental B Horlacher T Hershkovitz O De Paz JL Noti C. Natural cytotoxicity receptors NKp30, NKp44 and NKp46 bind to different heparan sulfate/heparin sequences. J Proteome Res. (2009) 8:712–20. 10.1021/pr800747c 19196184

[64] Hershkovitz O Jivov S Bloushtain N Zilka A Landau G Bar-Ilan A. Characterization of the recognition of tumor cells by the natural cytotoxicity receptor, NKp44. Biochemistry. (2007) 46:7426–36. 10.1021/bi7000455 17536787

[65] Zilka A Landau G Hershkovitz O Bloushtain N Bar-Ilan A Benchetrit F. Characterization of the heparin/heparan sulfate binding site of the natural cytotoxicity receptor NKp46. Biochemistry. (2005) 44:14477–85. 10.1021/bi051241s 16262248

[66] Higai K Imaizumi Y Suzuki C Azuma Y Matsumoto K. NKG2D and CD94 bind to heparin and sulfate-containing polysaccharides. Biochem Biophys Res Commun. (2009) 386:709–14. 10.1016/j.bbrc.2009.06.101 19555665

[67] Lai J-P Sandhu DS Yu C Moser CD Hu C Shire AM. Sulfatase 2 protects hepatocellular carcinoma cells against apoptosis induced by the PI3K inhibitor LY294002 and ERK and JNK kinase inhibitors. Liver Int. (2010) 30:1522–8. 10.1111/j.1478-3231.2010.02336.x 21040406 PMC3042145

[68] Pollari S Kakonen RS Mohammad KS Rissanen JP Halleen JM Warri A. Heparin-like polysaccharides reduce osteolytic bone destruction and tumor growth in a mouse model of breast cancer bone metastasis. Mol Cancer Res. (2012) 10:597–604. 10.1158/1541-7786.MCR-11-0482 22522458

[69] Cole CL Rushton G Jayson GC Avizienyte E. Ovarian cancer cell heparan sulfate 6-O-sulfotransferases regulate an angiogenic program induced by heparin-binding epidermal growth factor (EGF)-like growth factor/EGF receptor signaling. J Biol Chem. (2014) 289:10488–501. 10.1074/jbc.M113.534263 24563483 PMC4036170

[70] Nagarajan A Malvi P Wajapeyee N. Heparan sulfate and heparan sulfate proteoglycans in cancer initiation and progression. Front Endocrinol. (2018) 9:1–11. 10.3389/fendo.2018.00483 30197623 PMC6118229

[71] Blackhall FH Merry CLR Davies EJ Jayson GC. Heparan sulfate proteoglycans and cancer. Br J Cancer. (2001) 85:1094–8. 10.1054/bjoc.2001.2054 11710818 PMC2375159

[72] Mahtouk K Hose D Raynaud P Hundemer M Jourdan M Jourdan E. Heparanase influences expression and shedding of syndecan-1, and its expression by the bone marrow environment is a bad prognostic factor in multiple myeloma. Blood. (2007) 109:4914–23. 10.1182/blood-2006-08-043232 17339423 PMC2268882

[73] Gohji K Hirano H Okamoto M Kitazawa S Toyoshima M Dong J. Expression of three extracellular matrix degradative enzymes in bladder cancer. Int J Cancer. (2001) 95:295–301. 10.1002/1097-0215(20010920)95:5<295::aid-ijc1051>3.0.co;2-a 11494228

[74] Ogishima T Shiina H Breault JE Tabatabai L Bassett WW Enokida H. Increased heparanase expression is caused by promoter hypomethylation and up-regulation of transcriptional factor early growth response-1 in human prostate cancer. Clin Cancer Res. (2005) 11:1028–36. 15709168

[75] Vlodavsky I Friedmann Y Elkin M Aingorn H Atzmon R Ishai-Michaeli R. Mammalian heparanase: gene cloning, expression and function in tumor progression and metastasis. Nat Med. (1999) 5:793–802. 10.1038/10518 10395325

[76] Vornicova O Boyango I Feld S Naroditsky I Kazarin O Zohar Y. The prognostic significance of heparanase expression in metastatic melanoma. Oncotarget. (2016) 7:74678–85. 10.18632/oncotarget.12492 27732945 PMC5342694

[77] Ogren S Lindahl U. Cleavage of macromolecular heparin by an enzyme from mouse mastocytoma. J Biol Chem. (1975) 250:2690–7. 804478

[78] Höök M Wasteson Å Oldberg Å. A heparan sulfate-degrading endoglycosidase from rat liver tissue. Biochem Biophys Res Commun. (1975) 67:1422–8. 10.1016/0006-291X(75)90185-0 1035

[79] Putz EM Mayfosh AJ Kos K Barkauskas DS Nakamura K Town L. NK cell heparanase controls tumor invasion and immune surveillance. J Clin Invest. (2017) 127:2777–88. 10.1172/JCI92958 28581441 PMC5490772

[80] Barondes SH Castronovo V Cooper DNW Cummings RD Drickamer K Felzi T. Galectins: a family of animal β-galactoside-binding lectins. Cell. (1994) 76:597–8. 10.1016/0092-8674(94)90498-7 8124704

[81] Teichberg VI Silman I Beitsch DD Resheff G. A β D galactoside binding protein from electric organ tissue of *Electrophorus electricus*. Proc Natl Acad Sci USA. (1975) 72:1383–7. 10.1073/pnas.72.4.1383 1055413 PMC432538

[82] Hirabayashi J Kasai K. The family of metazoan metal-independent β-galactoside-binding lectins: structure, function and molecular evolution. Glycobiology. (1993) 3:297–304. 10.1093/glycob/3.4.297 8400545

[83] Chiariotti L Salvatore P Frunzio R Bruni CB. Galectin genes: regulation of expression. Glycoconj J. (2002) 19:441–9. 10.1023/B:GLYC.0000014073.23096.3a 14758067

[84] Johannes L Jacob R Leffler H. Galectins at a glance. J Cell Sci. (2018) 131:jcs208884. 10.1242/jcs.208884 29717004

[85] Cummings RD Liu F-T Vasta GR Galectins. In: Varki A Cummings RD Esko JD Stanley P Hart GW Aebi M editors. Essentials of Glycobiology. New York, NY: Cold Spring Harbor (2017).

[86] Liu F-T Rabinovich GA. Galectins as modulators of tumour progression. Nat Rev Cancer. (2005) 5:29–41. 10.1038/nrc1527 15630413

[87] Alhabbab R Blair P Smyth LA Ratnasothy K Peng Q Moreau A. Galectin-1 is required for the regulatory function of B cells. Sci Rep. (2018) 8:2725. 10.1038/s41598-018-19965-z 29426942 PMC5807431

[88] Bonzi J Bornet O Betzi S Kasper BT Mahal LK Mancini SJ. Pre-B cell receptor binding to galectin-1 modifies galectin-1/carbohydrate affinity to modulate specific galectin-1/glycan lattice interactions. Nat Commun. (2015) 6:6194–206. 10.1038/ncomms7194 25708191

[89] Stillman BN Hsu DK Pang M Brewer CF Johnson P Liu F-T. Galectin-3 and galectin-1 bind distinct cell surface glycoprotein receptors to induce T cell death. J Immunol. (2006) 176:778–89. 10.4049/jimmunol.176.2.778 16393961

[90] Garín MI Chu NC Golshayan D Cernuda-Morollón E Wait R Lechler RI. Galectin-1: a key effector of regulation mediated by CD4 +CD25+ T cells. Blood. (2007) 109:2058–65. 10.1182/blood-2006-04-016451 17110462

[91] Perillo NL Pace KE Seilhamer JJ Baum LG. Apoptosis of T cells mediated by galectin−1. Nature. (1995) 378:736–9. 10.1038/378736a0 7501023

[92] Kim HJ Jeon HK Cho YJ Park YA Choi JJ Do IG. High galectin-1 expression correlates with poor prognosis and is involved in epithelial ovarian cancer proliferation and invasion. Eur J Cancer. (2012) 48:1914–21. 10.1016/j.ejca.2012.02.005 22386573

[93] Jung EJ Moon HG Bok IC Jeong CY Joo YT Lee YJ. Galectin-1 expression in cancer-associated stromal cells correlates tumor invasiveness and tumor progression in breast cancer. Int J Cancer. (2007) 120:2331–8. 10.1002/ijc.22434 17304502

[94] Abroun S Otsuyama KI Shamsasenjan K Islam A Amin J Iqbal MS. Galectin-1 supports the survival of CD45RA(-) primary myeloma cells *in vitro*. Br J Haematol. (2008) 142:754–65. 10.1111/j.1365-2141.2008.07252.x 18537967

[95] Mathieu V De Lassalle EM Toelen J Mohr T Bellahcène A Van Goietsenoven G. Galectin-1 in melanoma biology and related neo-angiogenesis processes. J Invest Dermatol. (2012) 132:2245–54. 10.1038/jid.2012.142 22622427

[96] Cherayil BJ Weiner SW Pillai S. The Mac-2 antigen is a galactose-specific lectin that binds IgE. J Exp Med. (1989) 170:1959–72. 10.1084/jem.170.6.1959 2584931 PMC2189532

[97] Farhad M Rolig AS Redmond WL. The role of Galectin-3 in modulating tumor growth and immunosuppression within the tumor microenvironment. Oncoimmunology. (2018) 7:1–8. 10.1080/2162402X.2018.1434467 29872573 PMC5980349

[98] Nangia-Makker P Honjo Y Sarvis R Akahani S Hogan V Pienta KJ. Galectin-3 induces endothelial cell morphogenesis and angiogenesis. Am J Pathol. (2000) 156:899–909. 10.1016/S0002-9440(10)64959-0 10702407 PMC1876842

[99] Sano H Hsu DK Yu L Apgar JR Kuwabara I Yamanaka T. Human galectin-3 is a novel chemoattractant for monocytes and macrophages. J Immunol. (2000) 165:2156–64. 10.4049/jimmunol.165.4.2156 10925302

[100] Fukumori T Takenaka Y Yoshii T Kim HRC Hogan V Inohara H. CD29 and CD7 mediate galectin-3-induced type II T-cell apoptosis. Cancer Res. (2003) 63:8302–11. 14678989

[101] Kouo T Huang L Pucsek AB Cao M Solt S Armstrong T. Galectin-3 shapes antitumor immune responses by suppressing CD8 T cells via LAG-3 and inhibiting expansion of plasmacytoid dendritic cells. Cancer Immunol Res. (2015) 3:412–23. 10.1158/2326-6066.CIR-14-0150 25691328 PMC4390508

[102] Wada J Kanwar YS. Identification and characterization of galectin-9, a novel β-galactoside-binding mammalian lectin. J Biol Chem. (1997) 272:6078–86. 10.1074/jbc.272.9.6078 9038233

[103] Zhou X Sun L Jing D Xu G Zhang J Lin L. Galectin-9 expression predicts favorable clinical outcome in solid tumors: a systematic review and meta-analysis. Front Physiol. (2018) 9:452–67. 10.3389/fphys.2018.00452 29765332 PMC5939667

[104] Irie A Yamauchi A Kontani K Kihara M Liu D Shirato Y. Galectin-9 as a prognostic factor with antimetastatic potential in breast cancer. Clin Cancer Res. (2005) 11:2962–8. 10.1158/1078-0432.CCR-04-0861 15837748

[105] Wang Y Sun J Ma C Gao W Song B Xue H. Reduced expression of galectin-9 contributes to a poor outcome in colon cancer by inhibiting NK cell chemotaxis partially through the Rho/ROCK1 signaling pathway. PLoS ONE. (2016) 11:1–19. 10.1371/journal.pone.0152599 27028892 PMC4814049

[106] Nobumoto A Oomizu S Arikawa T Katoh S Nagahara K Miyake M. Galectin-9 expands unique macrophages exhibiting plasmacytoid dendritic cell-like phenotypes that activate NK cells in tumor-bearing mice. Clin Immunol. (2009) 130:322–30. 10.1016/j.clim.2008.09.014 18974023

[107] Zhu C Anderson AC Schubart A Xiong H Imitola J Khoury SJ. The Tim-3 ligand galectin-9 negatively regulates T helper type 1 immunity. Nat Immunol. (2005) 6:1245–52. 10.1038/ni1271 16286920

[108] Ndhlovu LC Lopez-Vergès S Barbour JD Brad Jones R Jha AR Long BR. Tim-3 marks human natural killer cell maturation and suppresses cell-mediated cytotoxicity. Blood. (2012) 119:3734–43. 10.1182/blood-2011-11-392951 22383801 PMC3335380

[109] Ju Y Hou N Meng J Wang X Zhang X Zhao D. T cell immunoglobulin- and mucin-domain-containing molecule-3 (Tim-3) mediates natural killer cell suppression in chronic hepatitis B. J Hepatol. (2010) 52:322–9. 10.1016/j.jhep.2009.12.005 20133006

[110] Blix F Gottschalk A Klenk E. Proposed nomenclature in the field of neuraminic and sialic acids. Nature. (1957) 179:1088. 10.1038/1791088b0 13430805

[111] Varki A Schauer R Sialic acids and other nonulosonic acids. In: Varki A. Cummings RD Esko JD Stanley P Hart GW Aebi M, editors. Essentials of Glycobiology. New York, NY: Cold Spring Harbor (2017).

[112] Peri S Kulkarni A Feyertag F Berninsone PM Alvarez-Ponce D. Phylogenetic distribution of CMP-Neu5Ac hydroxylase (CMAH), the enzyme synthetizing the proinflammatory human xenoantigen Neu5Gc. Genome Biol Evol. (2018) 10:207–19. 10.1093/gbe/evx251 29206915 PMC5767959

[113] Varki A Letter to the glyco-forum: since there are PAMPs and DAMPs, there must be SAMPs? Glycan “self-associated molecular patterns” dampen innate immunity, but pathogens can mimic them. Glycobiology. (2011) 21:1121–4. 10.1093/glycob/cwr087 21932452 PMC3150115

[114] Julien S Adriaenssens E Ottenberg K Furlan A Courtand G Vercoutter-Edouart AS. ST6GalNAc I expression in MDA-MB-231 breast cancer cells greatly modifies their O-glycosylation pattern and enhances their tumourigenicity. Glycobiology. (2006) 16:54–64. 10.1093/glycob/cwj033 16135558

[115] Wang P-H Feng Li Y Juang C-M Lee Y-R Chao H-T Tsai Y-C. Altered mRNA expression of sialyltransferase in squamous cell carcinomas of the cervix. Gynecol Oncol. (2001) 83:121–7. 10.1006/gyno.2001.6358 11585423

[116] Varki A Kannagi R Toole B Stanley P Glycosylation changes in cancer. In: Varki A. Cummings RD Esko JD Stanley P Hart GW Aebi M, editors. Essentials of Glycobiology. New York, NY: Cold Spring Harbor (2017).

[117] Rodrigues E Macauley MS. Hypersialylation in cancer: modulation of inflammation and therapeutic opportunities. Cancers. (2018) 10:1–19. 10.3390/cancers10060207 29912148 PMC6025361

[118] Büll C Boltje TJ Balneger N Weischer SM Wassink M van Gemst JJ. Sialic acid blockade suppresses tumor growth by enhancing T cell-mediated tumor immunity. Cancer Res. (2018) 78:canres.3376.2017. 10.1158/0008-5472.CAN-17-3376 29703719

[119] Paul A Padler-Karavani V. Evolution of sialic acids: implications in xenotransplant biology. Xenotransplantation. (2018) 25:e12424. 10.1111/xen.12424 29932472 PMC6756921

[120] Pillai S Netravali IA Cariappa A Mattoo H. Siglecs and immune regulation. Annu Rev Immunol. (2012) 30:357–92. 10.1146/annurev-immunol-020711-075018 22224769 PMC3781015

[121] Avril T Floyd H Lopez F Vivier E Crocker PR. The membrane-proximal immunoreceptor tyrosine-based inhibitory motif is critical for the inhibitory signaling mediated by Siglecs-7 and−9, CD33-related Siglecs expressed on human monocytes and NK cells. J Immunol. (2004) 173:6841–9. 10.4049/jimmunol.173.11.6841 15557178

[122] Mason LH Gosselin P Fogler WE Ortaldo JR McVicar DW Anderson SK. Differential tyrosine phosphorylation of inhibitory versus activating Ly-49 receptor proteins and their recruitment of SHP-1 phosphatase. J Immunol. (1997) 159:4187–96. 9379012

[123] Houchins JP Lanier LL Phillips JH Niemi EC Ryan JC. Natural killer cell cytolytic activity is inhibited by NKG2-A and activated by NKG2-C. J Immunol. (1997) 158:3603–9. 9103421

[124] Falco M Biassoni R Bottino C Vitale M Sivori S Augugliaro R. Identification and molecular cloning of P75/Airm1, a novel member of the sialoadhesin family that functions as an inhibitory receptor in human natural killer cells. J Exp Med. (1999) 190:793–802. 10.1084/jem.190.6.793 10499918 PMC2195632

[125] Nicoll G Ni J Liu D Klenerman P Munday J Dubock S. Identification and characterization of a novel siglec, siglec-7, expressed by human natural killer cells and monocytes. J Biol Chem. (1999) 274:34089–95. 10.1074/jbc.274.48.34089 10567377

[126] Zhang JQ Nicoll G Jones C Crocker PR. Siglec-9, a novel sialic acid binding member of the immunoglobulin superfamily expressed broadly on human blood leukocytes. J Biol Chem. (2000) 275:22121–6. 10.1074/jbc.M002788200 10801862

[127] Belisle JA Horibata S Gubbels JAA Petrie S Kapur A Andre S. Identification of Siglec-9 as the receptor for MUC16 on human NK cells, B cells, and monocytes. Mol Cancer. (2010) 9:118. 10.1186/1476-4598-9-118 20497550 PMC2890604

[128] Daly J Duggan T Hu J Natoni A Sarkar S Ki L Targeting siglec-7 : a novel immunotherapeutic approach to potentiate the cytotoxic functions of natural killer cells against multiple myeloma. Blood. (2017) 130:1799 10.1182/blood.V130.Suppl_1.1799.1799

[129] Cohen M Elkabets M Perlmutter M Porgador A Voronov E Apte RN. Sialylation of 3-methylcholanthrene–induced fibrosarcoma determines antitumor immune responses during immunoediting. J Immunol. (2010) 185:5869–78. 10.4049/jimmunol.1001635 20956342

[130] Prescher H Frank M Gütgemann S Kuhfeldt E Schweizer A Nitschke L. Design, synthesis, and biological evaluation of small, high-affinity siglec-7 ligands: toward novel inhibitors of cancer immune evasion. J Med Chem. (2017) 60:941–56. 10.1021/acs.jmedchem.6b01111 28103033

[131] Tanida S Akita K Ishida A Mori Y Toda M Inoue M. Binding of the sialic acid-binding lectin, siglec-9, to the membrane mucin, MUC1, induces recruitment of β-catenin and subsequent cell growth. J Biol Chem. (2013) 288:31842–52. 10.1074/jbc.M113.471318 24045940 PMC3814777

[132] Lloyd KO Burchell J Kudryashov V Yin BWT Taylor-Papadimitriou J. Comparison of O-linked carbohydrate chains in MUC-1 mucin from normal breast epithelial cell lines and breast carcinoma cell lines: demonstration of simpler and fewer glycan chains in tumor cells. J Biol Chem. (1996) 271:33325–34. 10.1074/jbc.271.52.33325 8969192

[133] Wong NK Easton RL Panico M Sutton-Smith M Morrison JC Lattanzio FA Characterization of the oligosaccharides associated with the human ovarian tumor marker CA125. J Biol Chem. (2003) 278:28619–34. 10.1074/jbc.M302741200 12734200

[134] Kufe DW. Mucins in cancer: function, prognosis and therapy. Nat Rev Cancer. (2009) 9:874–85. 10.1038/nrc2761 19935676 PMC2951677

[135] Chauhan SC Kumar D Jaggi M. Mucins in ovarian cancer diagnosis and therapy. J Ovarian Res. (2009) 2:21. 10.1186/1757-2215-2-21 20034397 PMC2804676

[136] Gubbels JA Felder M Horibata S Belisle JA Kapur A Holden H. MUC16 provides immune protection by inhibiting synapse formation between NK and ovarian tumor cells. Mol Cancer. (2010) 9:11. 10.1186/1476-4598-9-11 20089172 PMC2818693

[137] Felder M Kapur A Rakhmilevich AL Qu X Sondel PM Gillies SD Connor J Patankar MS. MUC16 suppresses human and murine innate immune responses. Gynecol Oncol. (2019) 152:618–28. 10.1016/j.ygyno.2018.12.023 30626487 PMC8327469

[138] Sternlicht MD Werb Z. How matrix metalloproteinases regulate cell behavior. Annu Rev Cell Dev Biol. (2001) 17:463–516. 10.1146/annurev.cellbio.17.1.463 11687497 PMC2792593

[139] Shiomi T Lemaître V D'Armiento J Okada Y. Matrix metalloproteinases, a disintegrin and metalloproteinases, and a disintegrin and metalloproteinases with thrombospondin motifs in non-neoplastic diseases. Pathol Int. (2010) 60:477–96. 10.1111/j.1440-1827.2010.02547.x 20594269 PMC3745773

[140] Page-McCaw A Ewald AJ Werb Z. Matrix metalloproteinases and the regulation of tissue remodelling. Nat Rev Mol Cell Biol. (2007) 8:221–33. 10.1038/nrm2125 17318226 PMC2760082

[141] Alfandari D McCusker C Cousin H. ADAM function in embryogenesis. Semin Cell Dev Biol. (2009) 20:153–63. 10.1016/j.semcdb.2008.09.006 18935966 PMC2693894

[142] Egeblad M Werb Z. New functions for the matrix metalloproteinases in cancer progression. Nat Rev Cancer. (2002) 2:161–74. 10.1038/nrc745 11990853

[143] Rocks N Paulissen G El Hour M Quesada F Crahay C Gueders M. Emerging roles of ADAM and ADAMTS metalloproteinases in cancer. Biochimie. (2008) 90:369–79. 10.1016/j.biochi.2007.08.008 17920749

[144] Waldhauer I Steinle A. Proteolytic release of soluble UL16-binding protein 2 from tumor cells. Cancer Res. (2006) 66:2520–6. 10.1158/0008-5472.CAN-05-2520 16510567

[145] Waldhauer I Goehlsdorf D Gieseke F Weinschenk T Wittenbrink M Ludwig A. Tumor-associated MICA is shed by ADAM proteases. Cancer Res. (2008) 68:6368–76. 10.1158/0008-5472.CAN-07-6768 18676862

[146] Groh V Wu J Yee C Spies T. Tumour-derived soluble MIC ligands impair expression of NKG2D and T-cell activation. Nature. (2002) 419:734–8. 10.1038/nature01112 12384702

[147] Ferrari de Andrade L Tay RE Pan D Luoma AM Ito Y Badrinath S. Antibody-mediated inhibition of MICA and MICB shedding promotes NK cell–driven tumor immunity. Science. (2018) 359:1537–42. 10.1126/science.aao0505 29599246 PMC6626532

[148] Dustin ML Springer TA. Lymphocyte function-associated antigen-1 (LFA-1) interaction with intercellular adhesion molecule-1 (ICAM-1) is one of at least three mechanisms for lymphocyte adhesion to cultured endothelial cells. J Cell Biol. (1988) 107:321–31. 10.1083/jcb.107.1.321 3134364 PMC2115164

[149] Barber DF Faure M Long EO. LFA-1 contributes an early signal for NK cell cytotoxicity. J Immunol. (2004) 173:3653–9. 10.4049/jimmunol.173.6.3653 15356110

[150] Wang R Jaw JJ Stutzman NC Zou Z Sun PD. Natural killer cell-produced IFN-γ and TNF-α induce target cell cytolysis through up-regulation of ICAM-1. J Leukoc Biol. (2012) 91:299–309. 10.1189/jlb.0611308 22045868 PMC3290424

[151] Reina M Espel E. Role of LFA-1 and ICAM-1 in cancer. Cancers. (2017) 9:153. 10.3390/cancers9110153 29099772 PMC5704171

[152] Koyama S. Immunosuppressive effect of shedding intercellular adhesion molecule 1 antigen on cell-mediated cytotoxicity against tumor cells. Japan J Cancer Res. (1994) 85:131–4. 10.1111/j.1349-7006.1994.tb02072.x 7908284 PMC5919416

[153] Becker JC Dummer R Hartmann AA Burg G Schmidt RE. Shedding of ICAM-1 from human melanoma cell lines induced by IFN-gamma and tumor necrosis factor-alpha. Functional consequences on cell-mediated cytotoxicity. J Immunol. (1991) 147:4398–401. 1684377

[154] Fiore E Fusco C Romero P Stamenkovic I. Matrix metalloproteinase 9 (MMP-9/gelatinase B) proteolytically cleaves ICAM-1 and participates in tumor cell resistance to natural killer cell-mediated cytotoxicity. Oncogene. (2002) 21:5213–23. 10.1038/sj.onc.1205684 12149643

[155] Wu J Mishra HK Walcheck B. Role of ADAM17 as a regulatory checkpoint of CD16A in NK cells and as a potential target for cancer immunotherapy. J Leukoc Biol. (2019) 105:1297–303. 10.1002/JLB.2MR1218-501R 30786043 PMC6792391

[156] Jing Y Ni Z Wu J Higgins L Markowski TW Kaufman DS. Identification of an ADAM17 cleavage region in human CD16 (FcγRIII) and the engineering of a non-cleavable version of the receptor in NK cells. PLoS ONE. (2015) 10:e0121788. 10.1371/journal.pone.0121788 25816339 PMC4376770

[157] Zhu H Blum R Bjordahl R Gaidarova S Rogers P Lee TT. Pluripotent stem cell-derived NK cells with high-affinity non-cleavable CD16a mediate improved anti-tumor activity. Blood. (2019) 10.1182/blood.2019000621 31856277 PMC7005364

[158] Peng YP Zhang JJ Liang WB Tu M Lu ZP Wei JS. Elevation of MMP-9 and IDO induced by pancreatic cancer cells mediates natural killer cell dysfunction. BMC Cancer. (2014) 14:1–12. 10.1186/1471-2407-14-738 25274283 PMC4287420

[159] Bruno A Bassani B D'Urso DG Pitaku I Cassinotti E Pelosi G. Angiogenin and the MMP9-TIMP2 axis are up-regulated in proangiogenic, decidual NK-like cells from patients with colorectal cancer. FASEB J. (2018) 32:5365–77. 10.1096/fj.201701103R 29763380

[160] Albertsson P Kim MH Jonges LE Kitson RP Kuppen PJ Johansson BR. Matrix metalloproteinases of human NK cells. In vivo. (2000) 14:269–76. 10757086

